# CRISPR/Cas Systems in Genome Editing: Methodologies and Tools for sgRNA Design, Off‐Target Evaluation, and Strategies to Mitigate Off‐Target Effects

**DOI:** 10.1002/advs.201902312

**Published:** 2020-02-06

**Authors:** Hakim Manghwar, Bo Li, Xiao Ding, Amjad Hussain, Keith Lindsey, Xianlong Zhang, Shuangxia Jin

**Affiliations:** ^1^ National Key Laboratory of Crop Genetic Improvement Huazhong Agricultural University Wuhan Hubei 430070 P. R. China; ^2^ Institute of Nuclear and Biological Technologies Xinjiang Academy of Agricultural Sciences Urumqi Xinjiang 830091 P. R. China; ^3^ Department of Biosciences Durham University Durham DH1 3LE UK

**Keywords:** base editing, CRISPR/Cas systems, off‐target effects, PAMs, sgRNA

## Abstract

Life sciences have been revolutionized by genome editing (GE) tools, including zinc finger nucleases, transcription activator‐Like effector nucleases, and CRISPR (clustered regulatory interspaced short palindromic repeats)/Cas (CRISPR‐associated) systems, which make the targeted modification of genomic DNA of all organisms possible. CRISPR/Cas systems are being widely used because of their accuracy, efficiency, and cost‐effectiveness. Various classes of CRISPR/Cas systems have been developed, but their extensive use may be hindered by off‐target effects. Efforts are being made to reduce the off‐target effects of CRISPR/Cas9 by generating various CRISPR/Cas systems with high fidelity and accuracy. Several approaches have been applied to detect and evaluate the off‐target effects. Here, the current GE tools, the off‐target effects generated by GE technology, types of off‐target effects, mechanisms of off‐target effects, major concerns, and outcomes of off‐target effects in plants and animals are summarized. The methods to detect off‐target effects, tools for single‐guide RNA (sgRNA) design, evaluation and prediction of off‐target effects, and strategies to increase the on‐target efficiency and mitigate the off‐target impact on intended genome‐editing outcomes are summarized.

## Graphical abstract

Herein, the off‐target effects, types, mechanism, major concerns, and outcomes of off‐target effects in plants and animals are summarized. Moreover, methods to detect off‐target effects, tools for sgRNA design, evaluation and prediction of off‐target effects, and strategies to increase the on‐target efficiency and mitigate the off‐target impact on intended genome‐editing outcomes are summarized.

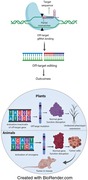

## 1 E‐CRISP/Cas Systems

Genome editing (GE) tools have modernized the genetics by their potential use in the precise modification of genomic DNA.^1^ These GE tools include ZFNs (zinc finger nucleases), TALENs (transcription activator‐like effector nucleases), CRISPR (clustered regulatory interspaced short palindromic repeats)/Cas9 (CRISPR‐associated proteins), and related CRISPR/Cas systems. Among these GE tools, CRISPR/Cas systems are extensively used in comparison with other methods because they are cost‐effective, easy to use, and do not require specialist skills.^2^ The CRISPR/Cas9 system requires an RNA designing with a short guide sequence (sgRNA) that directs a Cas9 nuclease for cleaving any target sequence.^3^ Cas9 is a CRISPR RNA‐guided endonuclease that cuts dsDNA targets complementary to the sgRNAs^4^ and is being exploited for GE in bacteria^5^ and in eukaryotic cells,^6^ including animal cells,^7^ mammalian systems,^8^, ^9^ and plants.^10^, ^11^

Base editing is a quite different and the most recent GE system, which has been widely used for introducing highly predictable and precise single nucleotide substitutions at genomic targets without requiring donor DNA templates, double‐strand breaks (DSBs) or dependence on homology‐directed repair (HDR) and non‐homologous end joining (NHEJ).^12^ Base editing technology is being used in various organisms and cell lines.^12^, ^13^ It has been considered more effective than HDR‐mediated base‐pair substitution.^13^ To date, several base editing systems, for instance BE3,^14^ BE4,^15^ Targeted‐AID,^16^ and dCpf1‐BE^17^ have been used in various organisms including major crops. These systems utilize Cas9 or Cpf1 systems for recruiting cytidine deaminases, which generate specific C–T alterations by using DNA mismatch repair pathways.^14^, ^15^, ^16^, ^17^

## 2 The Fidelity of CRISPR/Cas Systems

CRISPR/Cas9 nucleases have been used for GE in a wide diversity of living organisms, including major crops. Nevertheless, these GE systems can introduce unexpected off‐target mutations. Several reports revealed CRISPR/Cas9 system is more prone to off‐target effects than TALENs and ZFNs because it is a monomer, whereas the ZFN and TALEN assemblies are dimeric, facilitating identification of shorter target sequences. The Cas9 complex has been reported to bind to unintended regions and initiate cleavage, known as off‐target effects.^3^ Off‐targets are regions of gRNA that are highly homologous to the proposed on‐target regions. Normally off‐target regions have up to six mismatches compared with on‐target sites.^18^ Off‐targets with fewer mismatches have a tendency for more prominent binding and cleavage. However, a variety of tools are being developed for finding potential off‐target regions for given gRNA sequences, and so allow fewer mismatches.^18^ The off‐target effects of Cas9 were first studied in human cancer cell lines,^19^, ^20^, ^21^ where the frequency of those off‐target effects was remarkably high, because of incorrectly functioning DNA repair pathways in tumor cells.^22^

In base editing system, off‐target can result from gRNA dependent or gRNA independent editing events.^23^, ^24^, ^25^ A variety of strategies have been widely used for decreasing gRNA‐dependent off‐target base editing,^25^, ^26^ for instance the incorporation of mutations that enhance the specificity of DNA into the Cas9 component of base editors (BEs),^25^, ^27^, ^28^ adding 5′‐guanosine nucleotides to the sgRNA,^25^ or delivering the BEs as a ribonucleoprotein (RNP) complex.^25^, ^27^, ^28^ Off‐target editing based on gRNA‐independent arises by Cas9‐independent mannered binding to the deaminase domain of a BE to C or A bases.^23^, ^24^, ^26^

## 3 Types of Off‐Target Effects

Off‐target regions have previously been classified into three major types. The first includes regions at other PAMs (5′‐NGG‐3′) which have substitutions or mismatches.^20^, ^29^ The second type includes the regions at other PAMs (5′‐NGG‐3′) which contain insertions and/or deletions (indels) as comparison with target DNA or gRNA spacer.^30^ The DNA or RNA forms a small bulge with the residual nucleotides, and they correctly anneal, facilitating Cas9 activity. However, the off‐target activities detected at these sites are sometimes higher than on‐target activities.^30^ The third type regards the cutting of sequences with the different PAM sites (5′‐NAG‐3′).^20^, ^29^ However, it has also been proposed that there are two types of off‐target effects by CRISPR. The first type is the expected off‐targets in genomic regions which have high sequence similarity with the target. The second type includes the unexpected off‐targets in genomic regions which are not related to the target.^31^

## 4 Mechanism of Off‐Target Effects by CRISPR/Cas System

**Figure 1:**
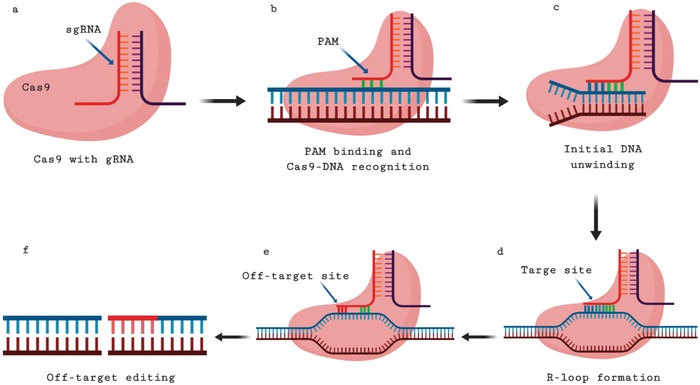
Mechanism of off‐target editing. CRISPR/Cas9 systems accept at least three mismatches in a 20 bp DNA target sequence. Cas9 may introduce unexpected off‐target mutations because sgRNAs recognize DNA sequences with one to a few nucleotide mismatches. a,b) PAM binding and recognition mediates the initial Cas9–DNA interaction, which leads to PAM‐proximal DNA melting. c) gRNA binding on target site initiates DNA unwinding. d) A stable R‐loop was formed between the crRNA and potential target sites. e) The sgRNA sequence recognizes partial mismatches outside the seed sequence instead of on‐target sites. f) Off‐target editing is generated as a result of mismatch recognition by sgRNA. Created with BioRender.com.

Accurate genome sequence information is essential for prediction of off‐target effects. Generally, the CRISPR/Cas system accepts no less than 3 mismatches in a 20‐base pair (bp) target DNA sequence. However, Cas9 may induce undesirable off‐target mutations because the sgRNAs recognize DNA sequences based on one to some nt mismatches, albeit with decreased binding and cleavage activity of the nuclease.^6^, ^29^, ^32^ The initial interaction between Cas9 and DNA is mediated by recognition and the binding at PAM site, which sequentially enables the melting of PAM‐proximal DNA and allows the directed probing of combining between the crRNA and the potential target editing sequences, forming a stable R‐loop.^33^, ^34^, ^35^ The binding and cleavage show different complementarity requirements within the 20 bp target. High stable binding needs however seven to nine matched bases in PAM‐proximal regions. It has been shown that as few as four mismatches in the PAM‐distal end hinder cleavage but not binding.^36^, ^37^ CRISPR/Cas9 specificity mainly relies on the sgRNA seed sequence within 10–12 bp directly 5′ of the NGG PAM‐proximal region.^38^ When the sgRNA sequence recognizes partial mismatches outside the seed sequence instead of on‐target sites, then off‐target edits will be produced.^39^ DNA bubbles are involved in inducing binding and cleavage of off‐target sequence at these sites. The partly unwound or melted DNA helps in recruiting Cas9 for binding and cleaving formerly hidden cryptic off‐target sites.^40^ The mechanism of off‐target editing by the CRISPR/Cas system is illustrated in **Figure** **1**.

### 4.1 Reasons for Off‐Target Effects

The targeting fidelity of Cas9 is considered to be highly determined by the 20 nt sgRNA and the PAM sites adjacent to the target sequence of the genome. However, the off‐target DNA cleavage will still happen on DNA sequences with up to 3–5 bp mismatches in the PAM‐distal region of the sgRNA targeting sequence.^8^, ^20^ Moreover, the cleavage of off‐ and on‐target sites can be affected by different guide RNA (gRNA) structures.^38^ Furthermore, it has been suggested by the studies of crystal structure and experiments of single‐molecule DNA curtain that the PAM sites are crucial for the binding activity of Cas9; and the seed sequence directly adjacent to PAM which corresponds to crRNA complementary recognition sequence (at 3′ end), is also essential for subsequent binding of Cas9, formation of R‐loop and the nuclease activities in Cas9.^41^, ^42^, ^43^

Additionally, many reports on off‐targets report that the type of mismatch type and its distance from the PAM sequence have significant importance. This information enables the development of a variety of off‐target scoring methods, aiding the selection of gRNAs with off‐target predictions.^18^, ^44^ The generation of DSBs in off‐target locations can be occurred due to the binding of Cas9 protein to PAM‐like sequences and/or the binding of gRNA to sequences which are identical to the target site.

### 4.2 Major Concerns/Outcomes of Off‐Target Effects

**Figure 2:**
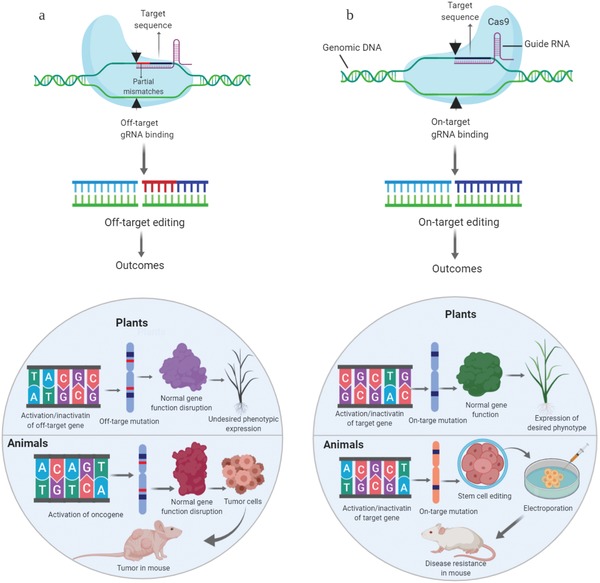
Major concerns/outcomes of off‐target effects. CRISPR/Cas systems usually offer great potential in genome editing, but off‐target activity, causing unintended consequences, is limiting its applications for therapeutic and agricultural purposes. a) CRISPR/Cas9 with a specific sgRNA may sometimes bind and edit at a site other than its target sequence, known as off‐target editing. This may result in unexpected serious consequences, such as the activation/inactivation of off‐target genes which can result in lethal or undesired phenotypes, or the activation of oncogenes causing cancer in animals. b) CRISPR/Cas9 that accurately edits its target gene is termed on‐target editing. CRISPR/Cas9 has been used in wide range of plants and animals due to its robust on‐target editing efficiency. On‐target editing leads to desired targeted phenotypes. Created with BioRender.com.

CRISPR/Cas systems show great potential in GE, but their off‐targeting may cause severe problems for the host organisms. Off‐targeting can lead to chromosomal rearrangements, causing damage at imperfectly matched genomic loci and limiting GE application for therapeutic purposes.^45^, ^46^ As well as interfering with chromosome stability, off‐target effects may cause loss of functional‐gene activity that causes diverse physiological or signaling abnormalities^29^, ^47^ (**Figure** **2**). It is therefore vital to design an optimum sgRNA for achieving high on‐targeting with no or little possibility for off‐target effects.^48^

#### 4.2.1 In Plants

CRISPR/Cas9 has been used for successful editing of more than 25 plant species and 100 genes to create variety of desirable traits in major crops.^49^, ^50^ However, the potential off‐target effects are the recent concern for the applications of CRISPR/Cas9 in plant,^51^ and this problem must be addressed if the technology is to be adopted for extensive use in gene therapy and crop breeding.^52^, ^53^, ^54^ Unlike gene therapy and clinical research in humans, research in plants is free from the same ethic issues, and there may be a higher tolerance for off‐target GE effects. It is critical to validate the phenotypic variations of interest for basic plant research. However, crop molecular breeding enables the elimination of off‐target mutations or spontaneous mutations causing inferior traits, via the selection of phenotypes during the breeding process. Therefore off‐target effects do not pose a major problem for practical crop breeding.^55^, ^56^

#### 4.2.2 In Animals

Unforeseen off‐target edits can be generated when the CRISPR/Cas systems do not cleave only the target region, and may cause potentially harmful effects, for example by activation of oncogenes.^57^, ^58^ As mentioned above, off‐target mutations may cause genome instability and disrupt gene function.^43^ The sgRNA‐dependent and ‐independent events can affect the overall stability of genomes in edited cells. For instance, sgRNA‐independent abnormalities, such as transduction of Cas9‐sgRNA complexes, can be induced by different kinds of stresses generated in different experimental conditions.^58^ Off‐targeting is a primary concern when applying CRISPR/Cas systems to clinical and biomedical applications.

## 5 Methods/Techniques to Detect Off‐Target Effects

A variety of in vitro bulk assays, single‐molecule, computational, and crystallography approaches are being used to understand the mechanism of binding and cleaving of targets by the Cas9 complex.^33^, ^34^, ^35^, ^36^, ^37^, ^40^, ^41^, ^60^ Different kinds of methods are used to detect and quantify off‐target effects caused by CRISPR/Cas9 and other CRISPR/Cas systems, and are described below (**Table** **1**).

**Table 1:** Methods to detect off‐target effects in living organisms

Method	Description	Uses	Limitation	Genome/Cell lines	Off‐target detection frequency	Refs.
T7E1 assay	An endonuclease involves in the cleavage of heteroduplexes formed by hybridization mutant DNA and WT sequences	Simple	Expensive and sensitivity is poor	–	–	9
Whole exome sequencing	This approach detects on‐ and off‐target mutations in the exome	Cheaper than WGS and has the ability to detect in unbiased manner mutations in coding regions	Depending on the organism, it does not detect mutations in the noncoding or regulatory regions, for instance, introns. May only cover a small percentage of the genome, depending on the organism	Human	–	22
WGS	WGS offers an unbiased and direct option for the assessment of mutations	A useful technique to analyze clones, single cells and F1 generation genome‐edited organisms	Only detects off‐target sites with higher frequency. It does not have the sensitivity required for detection of off‐target sites in bulk populations	Various living organisms	–	63
Deep sequencing	It detects indels at on‐and off‐target sites. It measures off‐target mutations that occur at 0.01% to 0.1%	Precise	A biased technique and does not have the ability to detect potential off‐target sites	–	–	133
ChIP‐seq	ChIP‐seq identifies the genome‐wide sgRNA:dCas9 binding sites	Detects genome‐wide Cas9 binding sites in an unbiased manner	Recognizes off‐target DNA‐binding sites by dCas9	–	–	134
GUIDE‐seq	Detects DSBs caused by nuclease activity on the basis of dsODNs integration into DSBs by NHEJ	Is sensitive, identifies translocations and breakpoint hotspots in an unbiased manner	Considerable false negatives and the efficiency is affected by chromatin accessibility	–	–	29
LAM‐HTGTS	Tracks genomic translocations caused by end‐joining between genomic DSBs. Detects DSBs caused by SSNs in a robust, sensitive and an unbiased manner	Detects DSBs and translocations	False negatives present and the efficiency is limited by chromatin accessibility	Human genome	–	120
Digenome‐seq	Widely used for genome‐wide profiling of off‐target effects	Detects off‐target sites with 0.1% or lower indel frequency in an unbiased manner	Has not been used extensively, is relatively expensive, especially when testing one gRNA and it requires a reference genome. When testing several sgRNAs, sequencing depth can be challenging	–	–	66
Multiplex Digenome‐seq	Profiles the genome‐wide specificities of more than 11 CRISPR/Cas9 nucleases concurrently, reduces the cost and saves time	Captures various bona fide off‐target mutations on a genome‐wide scale which can be missed by other techniques where indels are below 0.1% induced frequencies	–	–	–	128
ChIP‐dCas9	An indirect method, assumes the occurrence of Cas9 at any locus can induce DSBs	Has the ability to detect in situ interaction between proteins and DNA	–	–	–	18, 135
FISH	An efficient fluorescence in situ hybridization (FISH)‐based method which detects and evaluates nonspecific integrations of a given plasmid	Quick	Less precise	–	–	136
BLESS	Direct in situ breaks labeling, enrichment with high‐throughput sequencing and streptavidin	Detects DSBs induced by Sce endonuclease, complex genome‐wide DSB landscapes, and telomere ends	To directly map DSBs genome‐wide. Only detects the DSBs present during labeling period. Requires a reference genome	Human and mouse cells	More than 2000 aphidicolin‐sensitive regions (ASRs) identified	67
CIRCLE‐seq	Effective in vitro screening approach has the ability to outperform existing biochemical cell‐based methods for identifying genome‐wide CRISPR/Cas9 off‐target mutations	Identifies those off‐target mutations which are associated with cell‐type‐specific SNPs	Limited detection sensitivity and only works in the specific cells that can be labeled with GUIDE‐seq dsODN tag	Human genomic DNA	94%	69
SITE‐Seq	A biochemical that identifies the cleavage sites of Cas9 in purified genomic DNA	Examines off‐target mutations in cells, measures frequency of mutation and functional cellular consequence	–	Human genome	–	68
IDLV	Detects off‐target mutations of the CRISPR/Cas9 system and other kinds of nucleases	A highly effective approach for entering the nucleus of target cells, include the difficult‐to‐transfect primary cells of human	Programmable and sensitive. Cannot capture many bona‐fide off‐target sites	–	1%	137
GOTI	For evaluation of off‐target mutations caused by CRISPR/Cas9, CBE3, ABE systems	Detects off‐target mutations. Examines the population of cells derived from a single gene‐edited blastomere	–	Mouse	–	24
EndoV‐seq	Method for investigation of ABE specificity genome‐wide, where in vitro deaminated genomic DNA is digested with EndoV before being subjected to WGS	Enables evaluation of both on‐target and off‐target deamination by ABE. Amenable to multiplexing and offers clues to how ABE specificity may be improved	–	–	–	75
DISCOVER‐Seq	Powerful, sensitive assay for unbiased identification of off‐target sites in cellular models and in vivo	Detects off‐targets in cellular models and in vivo upon adenoviral gene editing	–	Human cell, Mouse liver		71
VIVO	A highly sensitive strategy for robust detection of in vivo genome‐wide off‐target mutations caused by CRISPR/Cas system	VIVO has been used to determine whether CRISPR/Cas systems induce substantial off‐target mutations in vivo	–	Mouse liver	–	75

### 5.1 Whole‐Genome Sequencing (WGS)

WGS is an unbiased and direct method for assessing mutations. However, it can detect only small percentages of off‐targets from bulk cells subjected to GE, and is impractical for distinguishing single nucleotide variants (SNVs) from those of sequencing errors and those that are naturally occurring. Therefore, it is important to seriously investigate the genetic background before assessment of off‐targets.^59^ WGS has been applied for detection of off‐target mutations by Cas9 in a variety of plants, including *Arabidopsis*,^61^ rice,^62^ tomato^63^ and cotton.^64^ However, WGS has only the ability to detect higher frequency off‐target sites with high reliability, and lacks the sensitivity required for the detection of off‐target sites in bulk populations.^18^, ^65^ WGS has the potential for detecting all types of off‐target base editing in cells or whole higher organisms when performed on genomic DNA from many independent cells.

### 5.2 Genome‐Wide, Unbiased Identification of DSBs Enabled by Sequencing (GUIDE‐seq)

GUIDE‐seq detects DSBs caused by nuclease activity based on the integration of double‐stranded oligodeoxynucleotides (dsODNs) into DSBs sites mediated by NHEJ , followed by the tagged DNA fragments amplification and sequencing.^22^ Cellular assays, such as GUIDE‐Seq,^29^ test nuclease cutting in a cellular context but rely on the integration of an exogenous DNA oligo that is inefficient in primary cells and not applicable in vivo. Furthermore, the cotransfection of additional exogenous DNA would not currently be used in human therapy for ethical reasons, and may affect overall editing outcomes.^66^

### 5.3 Digested Genome Sequencing (Digenome‐seq)

Digenome‐seq is an unbiased, robust, cost‐effective, sensitive, and reproducible method to profile off‐target effects genome‐wide of Cas9 and other programmable nucleases. Digenome‐seq is based on DNA cleavage rather than binding and this method is applied in the whole genomic context. Notably, Digenome‐seq captures potential off‐target sites with an RNA/DNA bulge. The Digenome‐seq is sensitive enough for detection of off‐target level where the induced indels are of 0.1% or lower frequency, which is almost the limit of detection of high‐throughput sequencing strategy.^67^

### 5.4 Bless

BLESS is direct in situ Breaks Labeling, Enrichment on Streptavidin, and next‐generation Sequencing and has been used in mouse and human cells with different sequencing platforms and DSBs‐inducing agents. BLESS has widely been used for detection of DSBs induced by Sce endonuclease, complex genome‐wide DSB landscapes and telomere ends. The direct in situ labeling avoids the labeling of DSBs which are artificially formed during the extraction of genomic DNA; so minimizing the risk of false positives.^68^

### 5.5 SITE‐Seq

SITE‐Seq is a method based on biochemical strategy that identifies the tagged genomic DNA ends and selective enrichment by sequencing (SITE‐Seq) for identification of cleavage sites of Cas9 in purified genomic DNA. In SITE‐Seq, genomic DNA is digested with various sgRNP concentrations, from limitation to saturation, and therefore permits the recovery of low‐ and high‐cleavage‐sensitivity off‐target sites. SITE‐Seq has been involved in producing highly enriched sequencing libraries for sgRNP cleavage fragments that enable specificity profiling with minimal read depth, which is crucial for the implementation of SITE‐Seq as a high‐throughput guide selection tool.^69^

### 5.6 CIRCLE‐seq

CIRCLE‐seq is a rapid, accessible, comprehensive, and highly sensitive in vitro detecting strategy to identify off‐target mutations of CRISPR/Cas9 in a genome‐wide scale.^70^ CIRCLE‐seq makes use of next‐generation sequencing technology, and has widely been used for the identification of off‐target mutations involved with cell‐type‐specific SNPs, which demonstrates the importance and feasibility for the generation of personalized specificity profiles.^69^ Moreover, CIRCLE‐seq could be applied for the detection of off‐target sites without presence of a reference genome or organisms lacking full genomic sequence availability.^70^

### 5.7 DISCOVER‐Seq

DISCOVER‐Seq is a powerful, sensitive assay for an unbiased identification of off‐target sites in cellular models. For example, it has been used in vivo following adenoviral gene editing of mouse livers, paving the way for real‐time discovery of off‐targets during therapeutic gene editing. DISCOVER‐Seq has been used with various types of Cas nucleases. DISCOVER‐Seq reliably detects off‐target editing from a variety of gRNAs, multiple Cas nucleases, in human and mouse cells^71^ and so potentially other species with large genomes.

### 5.8 GOTI

GOTI is an approach that evaluates the off‐target effects which are induced by CBE3, ABE 7.10, and CRISPR/Cas9.^23^, ^72^ GOTI has been used for the detection of off‐target mutations of mouse embryos at early stage by using either BEs or CRISPR/Cas9. Moreover, GOTI can evaluate off‐targets in the cell population derived from a single gene‐edited blastomere. GOTI could be useful for examining the off‐target effects of various gene‐editing tools without the interference of SNPs present in different individuals.^24^

### 5.9 EndoV‐seq (Endonuclease V Sequencing)

EndoV‐seq is a method widely used for the investigation of genome‐wide specificity of ABE, where Endonuclease V (EndoV) digests in vitro deaminated genomic DNA before being subjected to WGS. EndoV‐seq has been involved in the evaluation of both on‐ and off‐target deamination by ABE, and offers some clues to improve the specificity of ABE. EndoV‐seq has been involved in utilizing EndoV (deoxyinosine 3′ endonuclease) in vitro for nicking the DNA strand containing the inosine which is deaminated by ABE. The processed samples are then subjected to WGS for identification of off‐target sites.^73^ However, the specificity of BE and off‐target assessment needs an endonuclease for recognition of base I, the deaminated product of base A. EndoV from *Thermotoga maritimais* is a repair enzyme that has the ability to recognize the deoxyinosines and hydrolyze the second phosphodiester bond 3′ of the inosine base that results in nicked DNA.^74^

### 5.10 VIVO (Verification of in Vivo Off‐Targets)

VIVO is a very sensitive in vivo method which robustly detects off‐target effects caused by CRISPR/Cas nucleases in a genome‐wide scale.^75^ VIVO has been tested in vivo where a gRNA was deliberately designed with random sequence to check whether Cas9 can induce bona fide off‐targets in mouse livers. VIVO demonstrated that correctly designed gRNAs can direct the efficient in vivo editing in mouse livers without any detectable off‐target mutations. VIVO is therefore suitable to define and quantify the off‐target mutations of Cas9 proteins in whole organisms.^75^

## 6 Algorithms/Tools for sgRNA Target Finding and Evaluation, and Prediction of Off‐Target Effects

A variety of experimental systems are widely being used to investigate the off‐target effects of sgRNAs and they show different outcomes regarding the extent of off‐targets.^76^ Many algorithms have been applied for the prediction of off‐target effects and many Cas9 proteins with high specificity have successfully been produced for minimizing Cas9 promiscuity.^37^, ^40^, ^44^, ^77^, ^78^, ^79^, ^80^, ^81^, ^82^ However, sequencing‐based approaches have been widely used to experimentally validate a variety of Cas9 off‐targets that are not fully explained by such algorithms,^29^, ^67^ raising an interesting question about mechanisms of binding of Cas9 complexes and cleavage of off‐targets.^40^ Many tools have been developed and widely used on the basis of mismatch information, including the number and location of the mismatches, finding and evaluating the potential off‐target sites. Several groups have used these algorithms for defining potential off‐target sites (**Table** **2**).

**Table 2:** Algorithms for detection of off‐target effects

Algorithm	Description	Web source	Refs.
PEM‐seq	Detection of off‐target effects. Simultaneously determines the editing efficiency and specificity of CRISPR/Cas9	–	46
CRISPR‐PLANT v2	CRISPRPLANT v2 detects every off‐target	https://www.genome.arizona.edu/crispr2/	83
CCTop	Employs position‐dependent weight coefficients in their off‐target scoring algorithms	https://crispr.cos.uni-heidelberg.de	79
CROP‐IT	Scoring potential off‐target sites by the division of protospacer into three segments with weight coefficients optimized/rained with ChIP‐Seq data	https://www.adlilab.org/CROP-IT/homepage.html	85
CHOPCHOP	Rapid and easy selection of the optimal CRISPR/Cas9 or TALEN target sequences in genes from various organisms	https://chopchop.cbu.uib.no/, https://chopchop.rc.fasharvard.edu	86
CHOPCHOP v2	Web‐based tool for GE based on TALEN and CRISPR. A powerful and intuitive tool that serves both beginners and experienced users	https://chopchop.cbu.uib.no	138
CFD score	A scoring tool for a mismatch position and sequence‐dependent off‐target	https://www.broadinstitute.org/rnai/public/analysis-tools/sgrna-design https://research.microsoft.com/en-us/projects/azimuth/	78
Feng Zhang lab's Target Finder	Employs position‐dependent weight coefficients in their off‐target scoring algorithms	https://crispr.mit.edu/	18, 38
CT‐Finder	Predicts genomic off‐target sites	https://bioinfolab.miamioh.edu/ct-finder	87
CRISPOR	A web‐based tool which finds gRNAs in an input sequence and ranks them according to different scores evaluating potential off‐targets in the genome and predicts on‐target activity	https://crispor.org	44
CRISPR‐GE	Expedites experimental design and analyzes mutations for genome editing based on Cpf1/CRISPR/Cas9 in various organisms including plants	https://skl.scau.edu.cn/	89
An ensemble learning method	Predicts the off‐target sites of sgRNAs	https://github.com/penn‐hui/OfftargetPredict	48
Cas‐OFFinder	Searches the potential off‐target sites in user‐defined sequences or given genome	https://www.rgenome.net/cas-offinder/	77
MD	Analysis of the molecular function of CRISPR/Cas9	–	139

### 6.1 PEM‐seq (Primer‐Extension‐Mediated Sequencing)

PEM‐seq has been developed and widely used to detect off‐target effects and determination of specificity and editing efficiency of CRISPR/Cas9.^46^ LAM‐HTGTS has been combined with targeted sequencing by PEM‐seq for the effective analysis of CRISPR/Cas9 induced off‐target mutations via translocation capture. PEM‐seq can characterize off‐target sites and other abnormal chromosomal structures, such as genome‐wide translocations, small indels, and large deletions induced by Cas9. Furthermore, PEM‐seq has been employed for testing a variety of extensively used methods which were developed for reducing Cas9 off‐target activity, whereby PEM‐seq can assess comprehensively the specificity and editing efficiency of CRISPR/Cas9. This could greatly help in choosing an appropriate GE strategy for given loci.^46^

### 6.2 CRISPR‐PLANT v2

CRISPR‐PLANT is a platform for helping researchers in designing and constructing specific gRNAs for CRISPR/Cas9‐mediated GE in plants. CRISPR‐PLANT v2 detects every off‐target, where other tools have failed in detecting a subset of hidden sequences. However, off‐target sequences with gaps were not detected by other tools.^83^ This is important because of the considerable off‐target activity by CRISPR/Cas9 at sites with one gap or one to three mismatches.^20^, ^29^, ^30^ Most of the tools use Bowtie, though Bowtie has been designed for mapping up to 1024 bases of sequence when only one hit is expected to exist in the target genome.^84^

### 6.3 CCTop (CRISPR/Cas9 Target Online Predictor)

CCTop has widely been used to provide an intuitive user interface with easily adjustable default parameters by the user. It has the ability to identify and rank all the candidate sgRNA target sequences in accordance with their off‐target score, and shows the entire output. CCTop has been widely applied in gene inactivation, HDR and NHEJ experiments. CCTop helps to rapidly and efficiently identify the high quality target sites.^79^ It has numerous options for providing the list of top candidates to both a beginner and the expert, with complete documentation and flexible options. Therefore, the user will be well informed to select the suitable target sites.^79^

### 6.4 CROP‐IT (CRISPR/Cas9 Off‐Target Prediction and Identification Tool)

CROP‐IT is a web‐based tool that has been widely used to perform improved site predictions of binding and cleavage of off‐target editing. CROP‐IT has the ability to integrate biological information in the genome wide scale from current Cas9 editing profile data base (binding and cleavage). CROP‐IT has been shown to outperform current computational algorithms for predicting the Cas9 binding and cleavage sites. CROP‐IT outputs scores and ranks the potential off‐targets, enabling the improvements in the prediction of Cas9 editing profile and then accurate gRNA design.^85^

### 6.5 CHOPCHOP

CHOPCHOP is a web‐based tool that has been developed to accept numerous inputs (pasted sequences, genomic regions, gene identifiers) and provides a range of options to select any target. CHOPCHOP uses algorithms with efficient sequence alignment for minimizing search times, and prediction of off‐target editing of TALENs and CRISPR/Cas9. Additionally, for each potential target site, the primer candidates and restriction sites are visualized to facilitate an efficient pipeline for generation and validation of mutations. CHOPCHOP has been considered as a valuable tool for genome engineering, allowing users for rapidly and easily select the optimum target sequences for CRISPR/Cas9 or TALEN in genes from a wide range of organisms.^86^

### 6.6 CFD (Cutting Frequency Determination) Score

CFD score is a tool which has been widely used for scoring mismatch position and sequence‐dependent off‐targets with 240 fitting parameters.^78^ CFD score has been used to detect statistically significant increases in predicted off‐target sites for lethal sgRNAs. In addition, it has been suggested that the sgRNAs with higher off‐target risk are more easily improperly removed in the negative selection module. However, the avoidance of such uninhibited sgRNAs leads to improved library performance. CFD score predicts the probability of off‐target cutting and allows the decrease of majority of off‐target editing with high efficiency.^78^

### 6.7 CT‐Finder (CRISPR Target Finder)

CT‐Finder is web tool developed by Zhu et al. with multiple functions.^87^ Besides the routine service for the off‐target prediction for the CRISPR/Cas9 system, it also can be applied for the precise target prediction in two novel systems: RNA‐guided FokI nuclease (RFNs) and Cas9 D10A nickases (Cas9 n). Like other web tools, *CT* **‐** *Finder* can supports quite a few input setting including maximum numbers of gaps and numbers of mismatch as well as seed region length, with a very friendly and convenient visualization interface‐JBrowse, which can visualize off‐target and on‐target sequences in a genomic context, providing users the incorporation of user inputs for various important features, such as PAM sequence and gRNA length.

### 6.8 CRISPOR

CRISPOR, as a web tool, was developed by Concordet and Haeussler and designed for the gRNAs selection in a wide range genomes type. More than 150 genomes have been added into the data of this tool in the past 3 years. Once the candidate sgRNA sequences loading, CRISPOR will rank them with different scores regarding possible off‐target editing in the desired genome, and also to predict on‐target activity. It offers an inclusive solution from cloning, expression, and selection of gRNA and provides primers required for analysis of guide activity and possible off‐targets.^88^ CRISPOR ignores the candidate off‐target mutation with the off‐target score < 0.1 for the NGG PAM and those with a score less than 1.0 for NGA and NAG PAMs. CRISPOR currently supports 113 genomes.^44^

### 6.9 CRISPR‐GE

CRISPR‐GE is an integrated web‐based tool for expediting all experimental settings and analyses modifications by CRISPR/Cas/Cpf1 systems in various living organisms, including plants. It offers a robust toolkit for designing the target sgRNAs (targetDesign), primer design to construct the sgRNA expression cassettes, prediction of off‐target sites (offTarget) as well as the amplification of the target sites with genomic fragments (primerDesign). CRISPR‐GE provides a practical and comprehensive solution for GE in plants.^89^

### 6.10 An Ensemble Learning Method

Through this method, off‐target sites of a sgRNA from its thousands of candidates can be detected genome‐wide. This method is based on the occurrence of considerable differences in GC count and the preferences in a mismatch between the positive on‐target‐off‐target sequence pairs and those negative ones. This method enhances the efficiency of off‐target site prediction as compared to other computational methods, and can identify more off‐target sites consist with bona fide detections through high‐throughput methods. According to two case studies, it is efficient in the selection of optimal sgRNAs for treatment of certain genetic disorders.^48^

### 6.11 Cas‐OFFinder

Cas‐OFFinder is a versatile tool that detects possible off‐target mutation sites in a user‐defined sequence or a given genome. Compared with other tools for prediction of the off‐target sites of RGEN, Cas‐OFFinder has no limitations to the number of mismatches, allowing modifications in Cas9‐recognized PAM sequences, the vital protein component in RGENs. In any sequenced genome, Cas‐OFFinder allows rapid detection of potential off‐target editing without restraining the PAM sequence or the number of mismatched bases.^76^

## 7 Tools to Design Target‐sgRNA for GE

It is imperative to design sgRNA properly in order to eliminate lethal off‐targeting by GE tools. Various kinds of tools have been developed widely for designing sgRNAs with maximum accuracy (**Table** **3**).

**Table 3:** Tools to design target‐sgRNA

Tools	Description	Web source	Ref.
CRISPR‐P 2.0	Web service for computer‐aided designing of sgRNA with minimal chances of off‐target potentials	https://cbi.hzau.edu.cn/CRISPR2/	91
E‐CRISP	Web‐based application for designing gRNA sequences. Provides experiment‐oriented design and flexible output parameters that enables the design of many libraries	https://www.e-crisp.org/, https://www.e-crisp.org/E-CRISP/	92
Breaking‐Cas system	Web service for designing gRNAs for CRISPR/Cas9 system and other newly generated CRISPR/Cas systems available via ENSEMBL	https://bioinfogp.cnb.csic.es/tools/breakingcas	93
CasFinder	Extends and modifies a method to search the potential off‐targets for Cas9 by using queries that combine PAMs and seeds	https://arep.med.harvard.edu/CasFinder	18
CRISPR Design Tool	A computational tool, facilitating the selection and validation of sgRNAs and prediction of off‐target	https://www.genome-engineering.org/	38

### 7.1 CRISPR‐P 2.0

CRISPR‐P 2.0 is an online web tool providing services for designing sgRNA sequences and minimizing the possibilities of off‐target editing. It could design sgRNA for more than 50 plant genomes, including nearly all available major crop species (such as Rice, cotton, maize, wheat and so on) with high quality assembled genomes to date. It uses an adapted scoring method to rate the on‐target efficiency and off‐target potential of sgRNAs for SpCas9. The scoring method in CRISPR‐P 2.0 relies on recent research on the efficiency and specificity of SpCas9 in GE. CRISPR‐P 2.0 supports the designing of guide sequence for Cpf1 and other many CRISPR/Cas systems.^90^ It provides a comprehensive analysis of guide sequences, such as the GC content, secondary structure of sgRNA, sites for restriction endonucleases.^91^

### 7.2 E‐CRISP

E‐CRISP is an online application for designing gRNAs. It provides the identification of target sequences which are complementary to the gRNA ending in a 3′ PAM N(G or A)G that is essential for the recruitment of Cas9 nuclease to cleave dsDNA. It utilizes a fast indexing system to search a binary interval tree and binding sites for the rapid annotation of putative gRNA target sites. E‐CRISP also supports the reevaluation of CRISPR constructs for on‐ or off‐target sites as well as targeted genomic loci.^92^

### 7.3 Breaking‐Cas

Oliveros et al (2016) developed Breaking‐Cas System which is a tool to design gRNAs for CRISPR/Cas9 and other newly emerging variants. This tool offers unique features, possibly supports all eukaryotic genomes available in ENSEMBL and ENSEMBLGENOMES database. It is very flexible to identify and score the PAM motif sites (5′ or 3′) and its position. It also offers a valuable service for the assessing off‐targets of the gRNAs with the length of 18–25 nucleotides. Notably, the input form (providing a FASTA file) is very efficient and flexible (supporting the process of many sequences in a single run with multiple entries) , which is freely accessible on‐line like other tools.^93^

### 7.4 CasFinder

The CasFinder system was developed by Aach et al.,^94^ which revises the tool originally developed for identifying potential off‐targets generated by CRISPR/Cas9 system based on the combined querying for PAMs and seeds.^6^ This tool has several distinguishing features: this system offers flexible choosing in gRNA length, PAM sequence, number of mismatch and gaps; the JBrowse was introduced for the first time to visualize the off‐target and on‐target sites, which is very convenient and efficient for the user to evaluate the off‐targets effects within the genomic context. Notably, the CasFinder also supports the off‐target prediction for two newly emerging CRISPR/Cas variants RFNs and Ca9n, which are relied on the paired gRNAs and exhibited higher target specificity compared to the single CRISPR variant.

## 8 Strategies to Increase Target Editing Efficiency and Avoid or Minimize Off‐Target Editing of CRISPR/Cas System

Potential off‐target effects remain a major concern for many medical applications. Several efforts are being made to decrease off‐target activity of the CRISPR/Cas9 and for this purpose, high fidelity Cas9 variants and many other promising approaches are being developed for reducing possible off‐target activity, such as the optimization of sgRNA design,^95^ transcriptome analysis,^96^ and functional screening after dCas9 treatment.^97^ Additionally, off‐target effects can be reduced either by increasing the specificity of target site DNA/RNA cleavage mediated by nuclease e or by decreasing the duration of the nuclease expression for minimizing the chances of accumulation of off‐target mutations.^98^ Various other strategies and efforts include direct delivery (RNP complex),^99^, ^100^ separate Cas9 binding approaches (paired Cas9 nickases),^101^ truncated sgRNAs (small guide RNAs),^102^ and tunable systems (small‐molecule induction of Cas9, light‐activated and intein‐inactivated Cas9).^103^, ^104^, ^105^ Tunable or inducible systems regulate the Cas 9 working time, which is helpful decrease the undesirable DNA cleavage to the genome, which is one of major causes for the off‐target mutation. For example, the cleavage activity of Cas 9 could be blocked by two bacteriophage proteins AcrIIA2, AcrIIA4 (anti‐CRISPR proteins) after Cas9 cutting the target specific region.^106^ In recent years, serial Cas9 variants with high fidelity have been developed through optimizing and delineating the structure of Cas9 including eSpCas9(1.1), SpCas9‐HF1, Sniper‐Cas9, HypaCas9, xCas9(3.7), evoCas9, and SpCas9‐NG.^37^, ^80^, ^107^, ^108^, ^109^, ^110^

**Figure 3:**
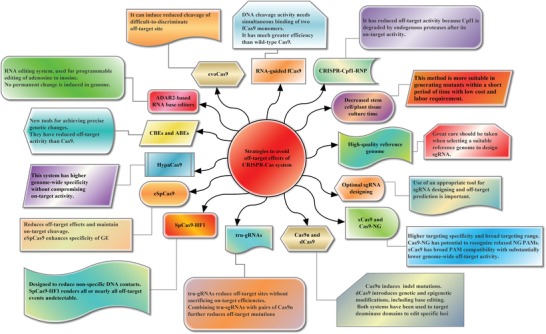
Several promising approaches have been adopted to decrease the off‐target activity of CRISPR/Cas systems, including decreased stem cell or plant tissue culture time, high‐quality reference genome, optimal sgRNA designing, and high‐fidelity CRISPR/Cas variants. These systems are engineered for greater precision, target specificity with no or at least much lower off‐target activity than wild‐type Cas9.

Off‐targets introduce ambiguity into scientific discoveries about the functions of any genes, and confounds potential therapeutic applications of CRISPR/Cas9 and other GE tools. The strategies to detect off‐target GE effects have been categorized into different groups, including mathematical and computational predication, validation of experimental off‐target cleavage, modification of Cas9‐sgRNA delivery, engineering of gRNA, and the engineering of high‐fidelity SpCas9.^111^ Different strategies to overcome or decrease off‐target effects are illustrated as follows and in **Figure** **3**.

### 8.1 Decreased Stem Cell or Plant Tissue Culture Time (to Avoid Somaclonal Variation)

Significant efforts are being made to understand the on‐ and off‐target editing for assisting the design of CRISPR systems with desirable efficiency. The prolonged timing for the stem cell or plant tissue culture means the longer duration of the nuclease expression, which will increase the risk of accumulation of off‐target mutations. Meanwhile, the somaclonal variations generated in the tissue culture and regeneration process will be dramatically accumulated and they will be the other kind of “off‐target” or unwanted mutations and have a more significant negative impact on the edited cell lines, plants than the off‐target editing mediated by nuclease. Therefore, it is very important to introduce any system which can reduce the time, cost, and labor in generating any transgenic mutant. Several systems, including tissue‐culture free systems, DNA‐free systems, transient systems, and VIGS‐mediated sgRNA delivery systems are likely to be suitable in generating mutants in shorter time, with low cost and reduced reliance on expert knowledge.

### 8.2 High‐Quality Reference Genome

Substantial progress has been made to avoid, and aid in the detection of genome‐wide off‐targets in an unbiased manner, and in the prediction of off‐target effects.^112^ These methods are often limited by their reliance on a reference genome for evaluation of efficacy, efficiency, and accuracy of gRNA. Current efforts have primarily focused on the improvement of the reference genome, and so great care must be taken in the selection of a suitable reference genome for designing sgRNAs. A recent report from our group regarding the off‐target effects of CRISPR‐Cas9 in cotton revealed that there has considerable genetic variation (up to 1 million SNPs and 14000 Indels) between the reference genome (cotton cultivar “TM‐1”) used for the sgRNAs design and the genome of the genotype used for genetic transformation (cotton cultivar “Jin 668” with higher plant regeneration ability). Our suggestion is that if you choose certain genotype to do genome editing, you should use the high‐quality genome of this genotype to design sgRNAs. Unfortunately, except the rice, Arabidopsis these model species, most genotypes in major crop species (cotton, maize, wheat, rapeseed, soybean, sorghum) used for genetic transformation do not have high‐quality genome sequences and the available reference genomes in these species are form different genotypes (Normally, these genotypes can't be used for plant tissue culture and genetic transformation because of low regeneration ability).^63^

### 8.3 Optimal sgRNA Design

Once high‐quality reference genomes for desired strains/cultivars/cells/lines are available, the following vital step is the selection of an appropriate tool to design sgRNAs and predict off‐targets. A variety of tools have been developed for designing sgRNAs which are being used to design optimum sgRNA sequences for minimum chances of off‐targeting, as discussed above.

### 8.4 Improved CRISPR/Cas Systems

#### 8.4.1 CBEs and ABEs (Cytosine and Adenine Base Editors, BEs) System for Base Editing

As introduced earlier, BEs are chimeric proteins made up of a catalytic domain and a DNA targeting modules with the capability to deaminate an adenine or a cytidine. These proteins do not require the generation of DSBs for editing of DNA bases, and therefore to decrease the occurrence of random indels at on‐target sites and mitigate the off‐target effects.^15^, ^27^ These are promising novel tools to obtain precise gene alterations necessary for trait improvement and disease treatment.^26^ CBEs and ABEs have been developed by fusing a nickase‐type Cas9 (nCas9) protein harboring a deaminase domain, which converts the C‐T (C>T) and A‐G, respectively, at the target site of a sgRNA.^23^, ^113^ However, ABEs allow the efficient and precise A‐T to G‐C base pairs conversion of targeted region within the editing window, generating minimal by‐products. ABEs have been shown to induce minimum DNA off‐target editing, whereas RNA off‐target editing with ABEs has not been studied in detail. ABE retains low DNA off‐targeting activity with decreased indel formation. Through decoupling the RNA and DNA editing activity, these ABE variants enhance the accuracy of adenine base editing via minimizing the off‐target editing activities of both RNA and DNA.^114^

In a study by Grünewald et al.,^115^ CBEs or ABEs were shown to be involved in inducing the guide‐RNA‐independent editing in a transcriptome‐wide scale of RNA bases. The selective inhibition of off‐target RNA editing (SECURE)‐BE3 systems was created to ensure reduction of unwanted RNA‐editing activity. The CBEs and ABEs have been found to exhibit the activity of RNA off‐target editing and have the ability to self‐edit their own transcripts, thus lead to the heterogeneity in the RNA sequences of BEs.^116^

Another study by Grünewald et al.^116^ showed the ability of CBE with rat APOBEC1 for causing the wide‐ranging cytosines deamination in human cells transcripts at the transcriptome‐wide scale which induces thousands of Cytidine‐to‐Uracil edits. CBE‐induced RNA edits have been found in noncoding and coding regions of proteins, and generating different types of mutations, such as nonsense, missense, splice site, and untranslated region mutations at 5′ and 3′. They also show that an ABE7 has the ability to induce transcriptome wide RNA edits.

Zhou et al.^117^ quantitatively evaluated single nucleotide variations (SNVs) that have been induced by CBEs or ABEs. The result show that these two systems can generate considerable off‐target SNVs in RNA sequences. Consequently, by engineering the deaminases, three CBE and one ABE variants have been found to reduce off‐target mutations in RNA SNVs while maintaining of an efficient on‐target activity with DNA.

#### 8.4.2 xCas9 and Cas9‐NG

xCas9 and Cas9‐NG are engineered versions of SpCas9, which show great potential to improve target specificity and extend target range. These Cas9 variants have been evaluated in the crop and model plant species, rice. xCas9‐3.7 is an efficient xCas9 variant showing targeted editing ability at up to 16 candidate NGN PAMs combinations such as NGG, NG, GAA, and GAT. xCas9 has been shown to have the broadest range of PAM recognition ability, such as GAT, GAA, and NG. xCas9 has various applications in human cells. Compared with SpCas9, xCas9 shows higher DNA specificity with considerably lower off‐target activity at all NGG PAMs on a genome‐wide scale, and the minimal off‐target activity at genomic regions with non‐NGG target sites.^118^ xCas9 has the ability to target canonical NGG PAMs while Cas9‐NG is a preferred enzyme to recognize relaxed PAMs for plant GE.^119^ The crystal structure of Cas9‐NG reveals that the newly added non‐base‐specific interactions can compensate the deficiency of base‐specific recognition with the third nucleotide base, which enable the recognition of NG PAM. It also introduces indels in human cells at endogenous targeting regions with NG PAMs.^110^

#### 8.4.3 Cas9n (Cas9 Nickase) and dCas9 (Dead Cas9)

Paired Cas9 nickase (Cas9n) is a mutated version of Cas9 where HNH or RuvC domains is inactivated by introducing a H840A or D10A alteration. Paired nickases are guided by two sgRNAs targeted to neighboring sites for inducing the offset nicks that generate the indel mutation.^98^, ^101^, ^120^, ^121^ Cas9 nickases have also been used as the targeting module that results in high frequencies of base editing.^101^, ^122^

The working model of dead Cas9 (dCas9) is very similar with Cas9n and has been to produce epigenetic and genetic modifications by fusing with different functional domains, such as single base mutations, to any specific DNA target sites. Since the base editing does not generate DSBs, both the Cas9 nickase and dCas9 have been utilized to edit specific loci by targeting the deaminase domains. Cytidine and adenine deaminases change their respective nucleotides (C and A) into T and G bases, which provide many opportunities for editing of any gene. Such base editing enzymes offer an excellent potential for their applications in basic biology, treatment of genetic disorders, and crop trait improvement.^123^

#### 8.4.4 Tru‐gRNAs (Shorter/Truncated Guide RNAs for On‐Target Site)

gRNAs with shortened length over the first 20 bp to 17/18 bp show reduced off‐target site modification without changing on‐target efficiency.^101^ For example, the use of truncated sgRNAs with a shortened 5′‐end of 17 or 18 nt significantly decreases undesirable mutagenic effects at off‐target sites in mammalian cell systems.^102^ Similarly, in plants, the use of truncated sgRNA in a CRISPR/Cas9 system using a constitutive promoter resulted in high on‐target mutation rates with no off‐target effects detected.^124^ Moreover, combining truncated sgRNAs with pairs of Cas9 nickase led to further reductions in off‐target mutations.^101^

#### 8.4.5 SpCas9‐HF1 (High‐Fidelity Engineered Variants of SpCas9)

SpCas9‐HF1 is a variant having high fidelity and is designed to decrease nonspecific DNA interactions. SpCas9‐HF1 has on‐target activity similar to WT SpCas9 and has been tested with > 85% of sgRNAs in human cells. This system preferentially interacts with typical nonrepetitive sequences has been observed to render most off‐target events, which is nondetectable by break capture and targeted sequencing strategy in a genome‐wide scale. Due to its extraordinary accuracy, SpCas9‐HF1 offers an alternate to WT SpCas9 for therapeutics and clinical applications.^80^

#### 8.4.6 eSpCas9 (Enhanced Specificity of SpCas9)

The eSpCas9 decreases off‐target activities and maintains efficient on‐target editing. The eSpCas9 can act as a highly useful for GE tool that require a high specificity such as clinical medicine. The enhanced specificity of GE could be obtained by reducing the binding with nontarget strand via a rationally generated eSpCas9 (K848A, K1003A, R1060A).^107^

#### 8.4.7 ADAR2‐Based RNA Base Editors

RNA BEs have been developed for modulating a range of biological processes by editing RNAs. For example, ADAR2 deaminates adenosine to inosine, which is read as guanine by the translational machinery.^125^ A recently repurposed RNA‐guided ribonuclease system uses CRISPR/Cas13 that enables the editing of mRNA sequences and adenosine to inosine editing by using a catalytically inactive Cas13 protein and the deaminase activity of ADAR2. This system, and similar other systems, have excellent potential to treat genetic diseases.^126^ However, the significant advantage of the application of RNA editing systems is that they do not introduce a permanent modification within the genome. Thus, these systems provide much better safety and ethical use compared to DNA base editing in humans.^125^ However, all the Cas13 proteins have the two enzymatically distinctive ribonuclease activities required for optimal interference.^127^

#### 8.4.8 CRISPR‐Cpf1‐RNP (Recombinant CRISPR‐Cpf1 Ribonucleoprotein)

The in vivo use of CRISPR‐Cpf1 has been found to exhibit decreased off‐target activity because that the Cpf1 will be degraded by endogenous proteases system in the target cells after Cpf1 performs its on‐target editing.^128^, ^129^ Nuclease concentration within the cell,^68^ delivery method (RNP vs plasmid)^68^, ^98^, ^130^ as well as more complex cellular properties, such as chromatin accessibility^131^, ^132^ have been shown to affect editing outcomes significantly and are generally missed by in vitro off‐target assays.

#### 8.4.9 HypaCas9 (Hyper‐Accurate Cas9 Variant)

HypaCas9 system enables a higher genome‐wide fidelity without affecting the on‐target genome editing in human cells. This system offers the potential as an improved approach to modify and rationalize the balance between nuclease activation and target recognition for precise GE.^37^

## 9 Concluding Remarks and Future Perspectives

A range of molecular tools are emerging for the GE of a wide range of living organisms, including animals, humans, and major plants species. These tools are proving themselves to be increasingly precise, efficient, effective, and reliable. CRISPR/Cas9 and other CRISPR/Cas systems are considered as revolutionary technologies that enable modifying nuclear genomes with unprecedented precision because of their accuracy, efficiency, cost‐effectiveness, and ease of use. Other CRISPR/Cas systems are being developed for efficient GE. However, GE can introduce a variety of off‐target mutations that can result in deleterious phenotypes, and so off‐target effects potentially limit the widespread use of GE. To overcome this problem, various systems are being developed to reduce off‐target effects, such as the newly generated CRISPR/Cas systems and the improvement of existing systems. Moreover, a range of techniques have been applied to detect off‐target mutations, and to improve the on‐target efficiency and decrease/avoid off‐target effects. In most cases, off‐target mutations can be ameliorated by specific sgRNAs selection with less predicted off‐targets, based on a robust reference genome sequence. After selection of the reference genome, it is critical to choose an appropriate tool for designing of sgRNAs and then the efficient delivery system (with the minimum level of somaclonal variations) of GE components into the target cells. Different software have been developed that can be used to design specific sgRNAs with negligible chances of off‐targeting, but more research is needed to further increase the on‐target specificity and avoid or mitigate the off‐target effect.

## Conflict of Interest

The authors declare no conflict of interest.
